# Race and in-hospital mortality after spontaneous intracerebral hemorrhage in the Stroke Belt: Secondary analysis of a case–control study

**DOI:** 10.1017/cts.2021.21

**Published:** 2021-03-16

**Authors:** Logan D. Hilton, Michael J. Lyerly, Toby I. Gropen

**Affiliations:** 1 School of Medicine, Louisiana State University, New Orleans, LA, USA; 2 Department of Neurology, University of Alabama at Birmingham, Birmingham, AL, USA

**Keywords:** Spontaneous intracerebral hemorrhage, stroke, ICH, race, disparities

## Abstract

**Background and Purpose:**

Intracerebral hemorrhage (ICH) accounts for around 10% of stroke, but carries 50% of stroke mortality. ICH characteristics and prognostic factors specific to the Stroke Belt are not well defined by race.

**Methods:**

Records of patients admitted to the University of Alabama Hospital with ICH from 2017 to 2019 were reviewed. We examined the association of demographics; clinical and radiographic features including stroke severity, hematoma volume, and ICH score; and transfer status with in-hospital mortality and discharge functional status for a biracial population including Black and White patients. Independent predictors of in-hospital mortality and functional outcome were examined using logistic regression.

**Results:**

Among the 275 ICH cases included in this biracial analysis, Black patients (*n* = 114) compared to White patients (*n* = 161) were younger (60.6 vs. 71.4 years, *P* < 0.0001), more often urban (81% vs. 64%, *P* < 0.01), more likely to have a history of hypertension (87% vs. 71%, *P* < 0.01), less often transferred (44% vs. 74%, *P* < 0.01), and had smaller median initial hematoma volumes (9.1 vs. 12.6 mL, *P* = 0.041). On multivariable analysis, Glasgow Coma Scale (GCS) for White patients (OR 13.0, *P* < 0.0001), hyperlipidemia for Black patients (OR 13.9, *P* = 0.019), and ICH volume for either race (Black patients: OR 1.05, *P* = 0.03 and White patients: OR 1.04, *P* < 0.01) were independent predictors of in-hospital mortality.

**Conclusions:**

Hypertension is more prevalent among Black ICH patients in the Stroke Belt. The addition of hyperlipidemia to the ICH score model improved the prediction of mortality for Black ICH patients. No differences in in-hospital mortality or poor functional outcome were observed by race.

## Introduction

Stroke is a leading cause of death and long-term disability in the USA [1]. Intracerebral hemorrhage (ICH), in particular, accounts for 10% of strokes but contributes to 50% of stroke mortality [2]. ICH risk factors in the US Stroke Belt population are not well characterized [3, 4]. This region consists of a group of Southeastern states including Alabama, Arkansas, Georgia, Indiana, Kentucky, Louisiana, Mississippi, North Carolina, South Carolina, Tennessee, and Virginia demonstrated in 1980 to have 10% higher stroke death rates than the US average [4]. Those living in the Stroke Belt have been noted to have more hypertension and diabetes mellitus [4, 5] along with lower socioeconomic status and less access to quality care [4]. The Stroke Belt population includes 27% non-Hispanic Blacks as compared to the national average of 10% [4]. As a result, this region is primarily biracial. Many prior studies focusing on racial disparities in this region have demonstrated disparities between those of Black and White race [4]. Improving ICH outcomes and reducing disparities in this region requires a clear understanding of the underlying associations with risk factors and outcomes.

In addition to geographic disparities, racial disparities in risk factors [6, 7] and outcomes [6] have been previously demonstrated in stroke patients [8]. A limitation of some previous studies examining disparities is that they often focus on an all-stroke prospective without specifically examining ICH [4]. Other studies have shown racial disparities in comorbidities [4], access to health care [9], and socioeconomic status [4] among ICH patients with limited emphasis on outcomes. Finally, while some studies have shown racial differences in both risk factors [3] and outcomes [10, 11] for ICH, these prior studies may be difficult to interpret due to variability in measurements of functional outcome. A study within the Stroke Belt is necessary to address the breadth and complexity of ICH and how it affects various outcome measurements.

Lastly, the influence of age, which carries a significant association with ICH etiology [12, 13], further compounds these considerations regarding ICH risk factors and outcomes. For example, while recent studies indicated poorer outcomes for White patients after ICH, these disparities were specific for younger adults and from a national perspective [14]. Within the Stroke Belt, greater incidence for ICH has been observed in Black patients for ages below 65, and White patients for ages above 65 [3]. As such, also examining ICH racial disparities within the context of age is necessary.

As age, geographic, and racial disparities have all been shown to affect risk factors, etiology, and outcomes, examining the influence of race on the complex nature of ICH requires a nuanced approach. The purpose of this biracial study is to examine potential race and age disparities for spontaneous ICH, as well as potential differences in the predictors for poor in-hospital outcomes based on race, within a Stroke Belt population.

## Methods

### Study Population

This is a secondary analysis of prospectively screened patients from a case–control study based on consecutive hospital admissions for bleeding events at a tertiary stroke center, the University of Alabama at Birmingham (UAB), between January 2017 and December 2019 (excluding a 5-month pause in enrollment from January to May 2018). Potential study participants included all bleeding-related admissions noted on the prospectively screened hospital admission list daily. Patients (≥18 years of age) with a major hemorrhage as defined using the International Society on Thrombosis and Hemostasis (ISTH) criteria were included [15]. From these screened major hemorrhages, intracranial hemorrhage events were selected in February 2020 for investigation.

A board-certified vascular neurologist (MJL) reviewed all patients admitted with intracranial hemorrhages in April and May 2020. We excluded patients with subarachnoid, subdural, and epidural hemorrhages, those with secondary intracranial hemorrhage (trauma, tumor, vascular lesion, secondary conversion of infarct, venous thrombosis, vasculopathy/vasculitis), or with major trauma. We included hemorrhages secondary to anticoagulant use unless a secondary etiology was identified. All cases not involving patients of the Black or White race were excluded to allow for direct comparisons between these two groups.

### Clinical Characteristics and Data Collection

Demographic information, medical comorbidities, social variables, admission and discharge medications, admission laboratory values, hospital management details, radiographic features, and in-hospital outcomes were gathered in February 2020 through retrospective chart review using an electronic health record system. Transfer status was defined as a transfer from other hospitals or freestanding emergency rooms. A measure of rurality was obtained by converting documented home zip code to Rural–Urban Commuting Area-2 (RUCA-2) codes [16]. RUCA-2 scores were dichotomized. A score of 1–3 was designated as urban, and a score of 4–10 was designated as rural, as micropolitan areas could be alternatively defined as large rural areas according to definition criteria [17]. The stroke etiology/mechanism was determined using neurology/neurosurgery attending documentation and independently reviewed by the board-certified vascular neurologist (MJL).

Clinical severity was defined using the National Institutes of Health Stroke Scale (NIHSS; scores of 0–42 with higher scores indicating higher degrees of stroke severity [18]) and ICH score (a clinical grading scale with scores of 0–6; higher scores reflecting greater stroke severity [19]) on admission were obtained from clinical documentation by trained healthcare providers. For clinical documentation of past medical history, missing data were assessed as indicating that the patient did not have the condition. For example, if no admission documentation mentioned a history of hypertension, then the patient was recorded as not having hypertension. In this way, no admission medical history data were defined as missing. Admission laboratory values were defined as the first value, if present, only within 24 h of admission. Information regarding mortality, discharge modified Rankin score (mRS), and discharge disposition were gathered from the inpatient hospital discharge note. The mRS is a 6-point disability scale with higher numbers indicating more severe disability and a score of 6 indicating death [20]. If the discharge mRS was not given in the provider discharge note (about 25% of the time), then it was determined based on the discharge physical exam, discharge inpatient physical therapy documentation, discharge inpatient occupational therapy documentation, and discharge nursing note.

For this analysis, we compared data based on the Black and White races. Our primary outcomes of interest were in-hospital mortality (defined as death from any cause during the singular inpatient hospital stay associated with the ICH event) and favorable discharge functional status (defined as an mRS score of 0–2). We also examined favorable discharge disposition which was defined as discharge home or to an inpatient rehabilitation facility, while poor discharge disposition was defined as the discharge to a skilled nursing facility, long-term acute care hospital, or death.

### Statistical Analysis

Statistical Analysis Software (SAS) version 9.4 was used. Mean and median values were created from quantitative variables for direct statistical comparison by race. Univariate and bivariate statistical analyses were performed to determine significant differences in the prevalence of risk factors between Black and White race subgroups, as well as rates of poor outcomes. These analyses included two-sided Student’s *t*-tests for continuous variables and chi-square analyses for categorical variables. To determine risk factors for in-hospital mortality, poor discharge mRS, or poor discharge disposition, logistic regression was performed. To assess confounding, variables significant on univariate analysis were then modeled in a stepwise fashion with other covariates plausibly associated with in-hospital mortality. Statistical significance was defined as a bidirectional alpha level of 0.05.

As the ICH score is frequently used to guide treatment and prognostication decisions, we sought to determine which items were most important in predicting in-hospital mortality from admission data. A multivariable model of admission data for the prediction of in-hospital mortality was created separately for each race. Variables previously shown to be associated with in-hospital mortality based on the ICH score, including Glasgow Coma Scale (GCS), infratentorial location, intraventricular hemorrhage, age greater than 80, and hematoma volume, were included in the model, in addition to the statistically significant variables for in-hospital mortality determined on our univariable analysis. Receiver operating characteristic (ROC) curves for these multivariable models were then created to examine the performance of these models.

## Results

**Fig. 1.**
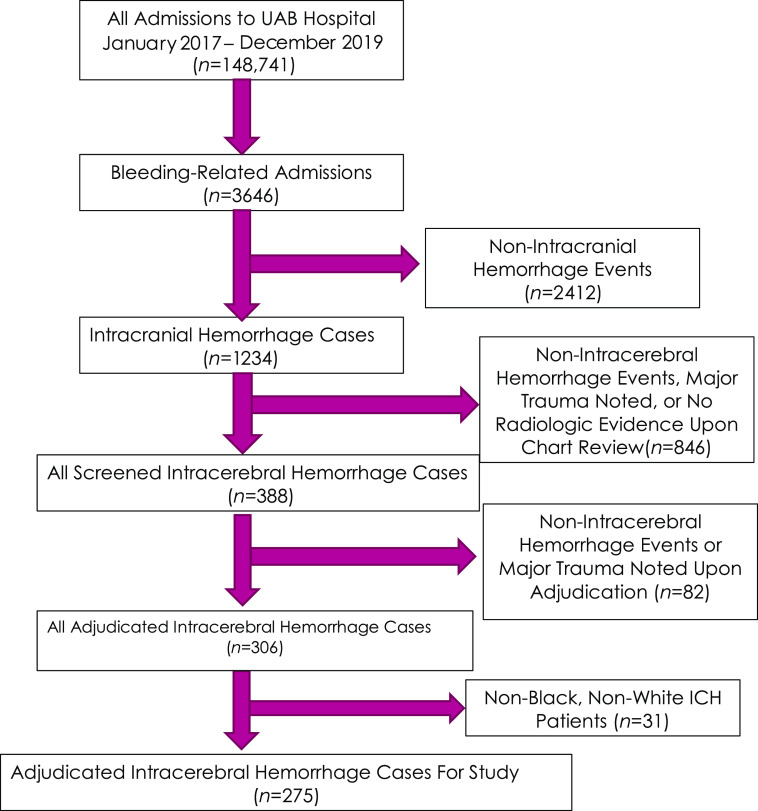
CONSORT diagram. ICH, intracerebral hemorrhage; UAB, University of Alabama at Birmingham.

A total of 148,741 admissions occurred during the study period, including 3647 bleeding events screened by hospital admission diagnosis, 1234 cases of intracranial bleeding, and 310 unique cases of spontaneous ICH. Cases of spontaneous ICH not involving patients of the Black or White race were excluded (Asian race [*n* = 6]; missing or unknown race [*n* = 29]). This resulted in 275 cases suitable for inclusion in the study (see CONSORT diagram, Fig. 1).

Patient characteristics are presented in Table 1. For the overall cohort, the median age was 66.5 years, 51% female, 41% Black, and 71% originating from an urban residence. Sixty-one percent of patients were transferred from other hospitals/freestanding emergency rooms. The majority of patients (52%) were diagnosed with a hypertensive hemorrhage, 10% were felt to be related to cerebral amyloid angiopathy (CAA), and 14% were related to anticoagulants. We observed a 31% in-hospital mortality rate, overall. Median discharge mRS was 4 with only 27% having a favorable discharge mRS of 0–2. Nearly half of the patients had a favorable discharge disposition to home or an inpatient rehabilitation facility, however. Our study also did not find differences in markers of ICH severity including initial hematoma volumes (*P* = 0.97) and ICH scores (*P* = 0.92) based on sex.

**Table 1.** Patient characteristics

Variable	Total (*n* = 275)	Black (*n* = 114)	White (*n* = 161)	*P*-value
Female	139 (51%)	61 (54%)	78 (48%)	
Age (years; mean ± STD)	66.5 ± 14.9	60.6 ± 14.6	71.4 ± 14.2	*P* < 0.0001
Urban (RUCA 1–3)	195 (71%)	92 (81%)	103 (64%)	*P* = 0.003
Etiology				
Hypertensive	142 (52%)	67 (59%)	75 (47%)	*P* = 0.13
Cerebral amyloid angiopathy	28 (10%)	8 (7%)	20 (12%)	
Cryptogenic/unknown	76 (28%)	27 (24%)	49 (30%)	
Anticoagulant-associated	39 (14%)	13 (11%)	26 (16%)	
Other (cocaine, cirrhosis, etc.)	8 (3%)	3 (3%)	5 (3%)	
Transfer status, Yes	169 (61%)	50 (44%)	119 (74%)	*P* < 0.01
Past medical history				
Hypertension	203 (77%)	94 (87%)	109 (71%)	*P* = 0.002
Diabetes	69 (26%)	28 (26%)	41 (27%)	*P* = 0.89
Hyperlipidemia	64 (24%)	23 (21%)	41 (27%)	*P* = 0.32
Congestive heart failure	32 (12%)	14 (13%)	18 (12%)	*P* = 0.76
Coronary artery disease	41 (16%)	12 (11%)	29 (19%)	*P* = 0.09
Atrial fibrillation	39 (15%)	11 (10%)	28 (18%)	*P* = 0.07
Chronic kidney disease	28 (11%)	12 (11%)	16 (10%)	*P* = 0.85
End-stage renal disease	18 (7%)	12 (11%)	6 (4%)	*P* = 0.02
Liver disease	8 (3%)	2 (2%)	6 (4%)	*P* = 0.35
Peripheral vascular disease	9 (3%)	3 (3%)	6 (4%)	*P* = 0.62
Dementia	23 (9%)	9 (8%)	14 (9%)	*P* = 0.85
Depression	20 (8%)	10 (9%)	10 (6%)	*P* = 0.41
Psychiatric conditions	30 (11%)	14 (13%)	16 (10%)	*P* = 0.52
Malignancy	29 (11%)	10 (9%)	19 (12%)	*P* = 0.43
DVT/PE	13 (5%)	7 (6%)	6 (4%)	*P* = 0.35
Ischemic stroke	11 (5%)	6 (6%)	5 (4%)	*P* = 0.34
Hemorrhagic stroke	18 (8%)	9 (10%)	9 (7%)	*P* = 0.39
Intracerebral hemorrhage	12 (5%)	5 (5%)	7 (5%)	*P* = 0.94
Admission medications				
Antiplatelet	29% (78)	30% (34)	28% (44)	*P* = 0.69
Aspirin	26% (70)	27% (31)	25% (39)	
Anticoagulant	17% (47)	16% (18)	18% (29)	*P* = 0.59
Statin	28% (76)	29% (33)	27% (43)	*P* = 0.75
Location by CT				
Lobar	96 (36%)	37 (32%)	59 (38%)	*P* = 0.36
Deep	174 (65%)	77 (68%)	97 (62%)	
Infratentorial	31 (11%)	9 (8%)	22 (14%)	*P* = 0.14
Intraventricular extension	120 (44%)	46 (40%)	74 (47%)	*P* = 0.31
Initial hematoma volume	10.9 ± 31.3	9.1 ± 26.3	12.6 ± 34.2	*P* = 0.041
ICH score	1.00 ± 1.44	1.00 ± 1.42	2.00 ± 1.47	*P* = 0.116
Admit NIHSS	8.00 ± 10.76	9.00 ± 10.9	9.00 ± 10.6	*P* = 0.68
Hospital course				
Intubation	95 (35%)	39 (34%)	56 (35%)	
Neurosurgical intervention	42 (15%)	17 (15%)	25 (16%)	
EVD placement	41 (15%)	18 (16%)	23 (14%)	
Anticoagulation reversal	28 (10%)	9 (8%)	19 (12%)	
Admission lab values				
Leukocyte count	9.27 ± 4.32	7.9 ± 3.5	10.7 ± 4.4	*P* < 0.0001
Platelets	214.9 ± 83.9	212.5 ± 76.7	216.3 ± 88.6	*P* = 0.22
Glucose	132.5 ± 65.4	129.0 ± 66.7	134 ± 64.6	*P* = 0.55
INR	1.05 ± 0.83	1.05 ± 1.00	1.05 ± 0.66	*P* = 0.41
Discharge mRS				
Favorable (0–2)	75 (27%)	30 (26%)	45 (28%)	*P* = 0.76
Poor (3–6)	200 (73%)	84 (74%)	116 (72%)	
Discharge mRS	4.00 ± 1.9	4.00 ± 1.9	4.00 ± 1.92	*P* = 0.31
In-hospital mortality	85 (31%)	29 (25%)	56 (35%)	*P* = 0.09
Discharge disposition				
Favorable	133 (49%)	64 (57%)	69 (43%)	*P* = 0.02
Poor	140 (51%)	48 (43%)	92 (57%)	

CT, computed tomography; DVT, deep venous thrombosis; EVD, external ventricular drain; ICH, intracerebral hemorrhage; INR, international normalized ratio; mRS, modified Rankin scale; NIHSS, National Institutes of Health Stroke Scale; PE, pulmonary embolism; RUCA, rural urban commuting area.

### Differences in ICH Characteristics and Risk Factors by Race

Racial differences in demographics, risk factor prevalence, admission lab values, radiologic data, and discharge disposition were observed among ICH patients (Table 1). Black patients were younger (60.6 vs. 71.4 years, *P* < 0.0001), more likely to reside in urban areas (81% vs. 64%, *P* < 0.01), and were less likely to have an interhospital transfer (44% vs. 74%, *P* < 0.01). Black patients more often had a previous diagnosis of hypertension. They also were admitted with lower median leukocyte count, as well as smaller median initial hematoma volume (9.1 vs. 12.6 mL, *P* = 0.041). No significant differences in admission NIHSS score or ICH score were observed based on race. Furthermore, no significant differences were observed with in-hospital mortality or poor discharge mRS, although there were significantly greater rates of favorable discharge disposition among Black patients (57% vs. 43%, *P* = 0.002). However, adjustment for initial hematoma volume attenuated this association.

Patients were further stratified to those below 65 (“young”) and those 65 and older (“old”) to describe the effect of age (Table 2). Across age groups, Black patients had a lower likelihood of interhospital transfer compared to White patients. Among the older population, Black patients more often originated from an urban residence and had higher rates of previous hypertension (98% vs. 74%, *P* < 0.01). We also observed that young Black patients had smaller hematoma volumes (8.5 vs. 11.1 mL, *P* = 0.03), but volumes did not significantly differ among older age groups. A lower rate of code status change to Do-Not-Resuscitate (DNR) for Black patients was also observed among the young cohort (7% vs. 23%, *P* = 0.01), while there was no significant difference in the older cohort.

**Table 2.** Patient characteristics stratified by race and age

Variable	Black <65	White <65	*P*-value	Black >65	White >65	*P*-value
	(*n* = 72)	(*n* = 53)		(*n* = 42)	(*n* = 108)	
Sex, Male	39 (54%)	32 (60%)	*P* = 0.49	14 (33%)	51 (47%)	*P* = 0.12
Age (years; mean ± STD)	53.5 ± 9.0	52.0 ± 9.0		74.1 ± 8.3	78.0 ± 7.2	
Urban (RUCA 1–3)	55 (76%)	33 (62%)	*P* = 0.09	37 (88%)	70 (65%)	*P* = 0.005
Transfer status, Yes	34 (47%)	41 (77%)	*P* < 0.001	16 (38%)	78 (72%)	*P* < 0.01
ICH score	1.0 ± 1.4	1.0 ± 1.5	*P* = 0.43	1.5 ± 1.4	2.0 ± 1.5	*P* = 0.49
Admit NIHSS	8.5 ± 11.4	7.0 ± 11.5	*P* = 0.67	11.0 ± 10.1	9.0 ± 10.3	*P* = 0.93
Hospital course						
Intubation	27 (38%)	24 (45%)	*P* = 0.38	12 (29%)	32 (30%)	*P* = 0.89
Neurosurgical intervention	14 (19%)	11 (21%)	*P* = 0.86	3 (7%)	14 (13%)	*P* = 0.31
EVD placement	15 (21%)	11 (21%)		3 (7%)	12 (11%)	
Anticoagulation reversal	5 (7%)	2 (4%)	*P* = 0.45	4 (10%)	17 (16%)	*P* = 0.32
Initial hematoma volume	8.5 ± 21.0	11.1 ± 33.4	*P* = 0.03	11.9 ± 33.6	13.2 ± 34.7	*P* = 0.48
Location						
Lobar	17 (24%)	15 (30%)		20 (48%)	44 (42%)	
Deep	55 (77%)	37 (74%)		22 (22%)	60 (57%)	
Intraventricular extension	33 (46%)	28 (55%)	*P* = 0.32	13 (31%)	46 (43%)	*P* = 0.66
Infratentorial	5 (7%)	9 (17%)	*P* = 0.08	4 (10%)	13 (12%)	*P* = 0.19
Code status changed to do-not-resuscitate	5 (7%)	12 (23%)	*P* = 0.01	8 (19%)	18 (17%)	*P* = 0.73
Discharge mRS						
Favorable (0–2)	21 (29%)	14 (26%)	*P* = 0.73	9 (21%)	31 (29%)	*P* = 0.37
Poor (3–6)	51 (71%)	39 (74%)		33 (79%)	77 (71%)	
In-hospital mortality	16 (22%)	20 (38%)	*P* = 0.06	13 (31%)	36 (33%)	*P* = 0.78
Discharge disposition						
Favorable	46 (66%)	31 (58%)	*P* = 0.41	18 (43%)	38 (35%)	*P* = 0.38
Poor	24 (34%)	22 (42%)		24 (57%)	70 (65%)	

EVD, external ventricular drain; ICH, intracerebral hemorrhage; mRS, modified Rankin scale; NIHSS, National Institutes of Health Stroke Score; RUCA, rural urban commuting area.

When compared solely on the basis of age (not race), young patients had higher rates of deep bleeds involving the basal ganglia, internal capsule, thalamus (*P* = 0.01), and hypertensive or cryptogenic etiologies (*P* < 0.01). When comparing young Black patients to old Black patients or young White patients to old White patients, both comparisons also showed higher rates of deep bleeds (*P* < 0.01 and *P* = 0.02, respectively) and hypertensive etiology (*P* = 0.02 and *P* < 0.01, respectively) among the young patients. Yet, further stratification by both race and age did not reveal significant differences in ICH location or etiology across races and ages. Finally, in-hospital mortality and functional outcome did not differ across races and ages.

### Predictors of In-Hospital Mortality

The results for univariate risk for in-hospital mortality based on logistic regression are shown in Table 3. For both Black and White patients, in-hospital mortality was predicted by admission leukocyte count, admission glucose level, admission NIHSS, admission ICH score, need for intubation, initial hematoma volume, intraventricular hemorrhage, spot sign on CT angiogram, and hematoma expansion on follow-up imaging. Black patients requiring intubation were at a 22-fold increased risk of in-hospital mortality, whereas White patients were at a 20-fold increased risk. When adjusted for initial hematoma volume, intraventricular extension, and ICH score, intubation remained a highly significant predictor of mortality. For only Black patients, in-hospital mortality was also predicted by the previous history of hyperlipidemia (admission total cholesterol), documented antiplatelet use, and neurosurgical intervention (including placement of external ventricular drain [EVD] for intracranial pressure monitoring). There were no variables that predicted in-hospital mortality for only White patients.

**Table 3.** Predictors of in-hospital mortality among patients with intracerebral hemorrhage (ICH)

	Black patients (*n* = 114)			White patients (*n* = 161)		
Variable	OR	95% CI	*P*-value	OR	95% CI	*P*-value
Age, per year	1.03	0.996–1.057	0.09	1.00	0.98–1.02	0.92
Sex, Male	1.10	0.47–2.56	0.82	0.73	0.38–1.39	0.34
Geography, Rural	0.83	0.28–2.50	0.74	1.24	0.63–2.43	0.53
Interhospital transfer	0.48	0.20–1.18	0.10	0.82	0.40–1.71	0.13
Comorbidities						
Prior stroke or transient ischemic attack	0.91	0.34–2.43	0.85	1.98	0.92–4.29	0.08
Hypertension	4.97	0.62–39.9	0.07	1.19	0.56–2.49	0.65
Diabetes mellitus	1.00	0.37–2.70	1.00	0.88	0.41–1.89	0.74
Hyperlipidemia	5.09	1.89–13.7	<0.001	0.88	0.41–1.89	0.74
Depression	0.73	0.15–3.67	0.70	0.47	0.10–2.29	0.32
Dementia	2.61	0.65–10.5	0.19	2.11	0.70–6.38	0.19
Psychiatric illness	0.80	0.21–3.1	0.74	0.88	0.29–2.68	0.82
Medications						
Antiplatelet use	3.03	1.25–7.34	0.01	0.76	0.36–1.61	0.46
Anticoagulant use	0.81	0.24–2.70	0.73	1.76	0.77–4.00	0.18
Statin use	1.75	0.72–4.28	0.22	0.91	0.43–1.91	0.79
Lab values						
Admit leukocyte count, per 1.0 × 10^3^ per µL	1.13	1.00–1.27	0.04	1.21	1.11–1.32	<0.001
Admit platelet count, per 1.0 × 10^9^/L	0.996	0.99–1.00	0.17	1.003	0.999–1.01	0.09
Admit glucose, per 1 mg/dL	1.007	1.00–1.01	0.03	1.01	1.01–1.02	<0.001
Admit prothrombin time	1.03	0.98–1.09	0.21	1.07	0.99–1.16	0.095
Admit INR	1.32	0.85–2.06	0.17	1.90	0.87–4.15	0.109
Admit total cholesterol, per 1 mg/dL	0.98	0.97–0.995	<0.01	0.995	0.99–1.00	0.29
Hospital course						
NIHSS, per 1 pt	1.18	1.11–1.26	<0.0001	1.2	1.13–1.27	<0.0001
ICH score, per 1 pt	3.80	2.29–6.32	<0.0001	3.99	2.60–6.12	<0.0001
Intubation	22.4	7.36–68.2	<0.0001	19.5	8.54–44.5	<0.0001
Neurosurgical intervention	4.33	1.48–12.7	<0.01	1.59	0.67–3.78	0.30
EVD placement	3.8	1.33–10.8	0.01	1.89	0.78–4.62	0.16
Reversal therapy	0.83	0.16–4.22	0.82	2.32	0.88–6.10	0.08
Radiologic data						
Initial hematoma volume, per 1 mL	1.05	1.02–1.07	<0.0001	1.05	1.03–1.07	<0.0001
Intraventricular hemorrhage	5.04	2.03–12.6	<0.001	6.66	3.19–13.88	<0.0001
Spot sign, if CT angiogram done (*n* = 162)	6.13	1.28–29.4	0.02	4.46	1.36–14.64	0.014
Hematoma expansion	3.93	1.05–14.7	0.042	21.5	4.43–104.1	<0.001
Location, Lobar	2.07	0.86–4.93	0.10	1.15	0.59–2.26	0.68
Location, Deep	0.57	0.24–1.36	0.20	1.10	0.56–2.17	0.78
Microhemorrhages	0.71	0.11–4.47	0.71	0.87	0.33–2.26	0.77
Deep	0.42	0.04–3.92	0.44	0.71	0.25–2.01	0.52
Cortical	1.04	0.11–10.03	0.98	1.48	0.52–4.15	0.46
>10	0.65	0.07–6.17	0.70	0.74	0.24–2.23	0.59
Superficial siderosis	4.19	0.38–46.7	0.24	1.83	0.43–7.79	0.41

CT, computed tomography; EVD, external ventricular drain; INR, international normalized ratio; NIHSS, National Institutes of Health Stroke Scale.

The unique risk factors for in-hospital mortality for Black patients were then examined further by multivariable analysis. After adjustment for initial hematoma volume, antiplatelet use remained significantly associated with mortality (OR 3.40, 95% CI 1.19–9.67, *P* = 0.02). When further adjusted for history of atrial fibrillation, congestive heart failure, hypertension, age greater than 65, diabetes mellitus, history of stroke or TIA, and peripheral vascular disease, the risk still remained significant (OR 3.60, 95% CI 1.03–12.6, *P* = 0.047). The influence of neurosurgical intervention was attenuated on adjustment for markers of severity including ICH score and initial hematoma volume.

**Fig. 2.**
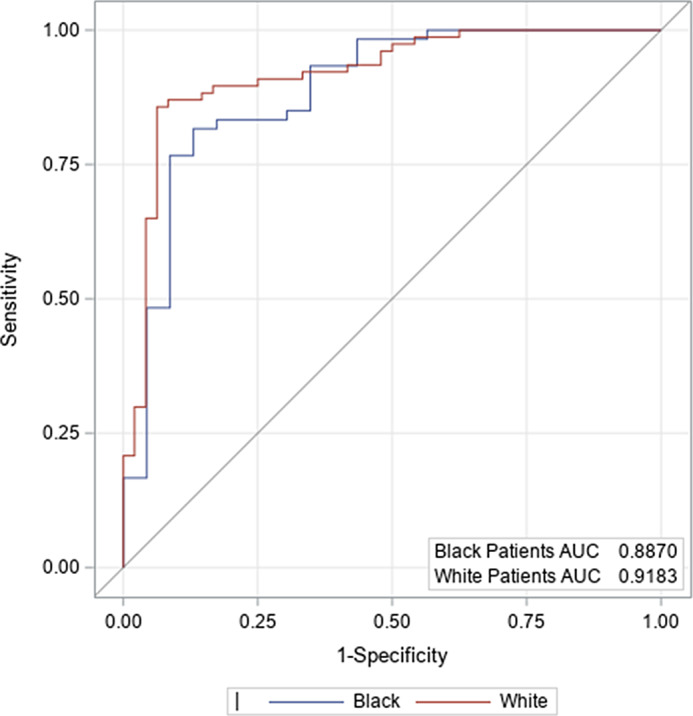
Receiver operating characteristic for in-hospital mortality based on intracerebral hemorrhage (ICH) score. AUC, area under the curve.

**Fig. 3.**
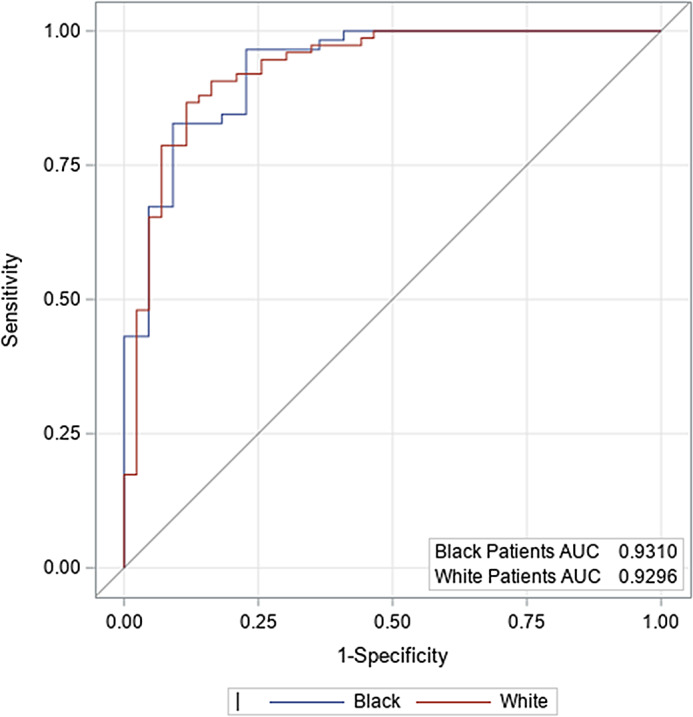
Receiver operating characteristic for in-hospital mortality based on multivariable model. AUC, area under the curve.

Multivariable models for in-hospital mortality were created based on ICH score components and variables significant for mortality for each race from univariable analysis (antiplatelet use, hyperlipidemia, leukocyte count, and glucose). For all patients, incremental increases in initial ICH volumes were associated with in-hospital mortality (Table 4). The admission GCS was a significant predictor among White patients (OR 11.8 for each category jump within the ICH score, 98% CI 3.95–35.13, *P* < 0.0001), but not among Black patients. Presence of intraventricular hemorrhage, age greater than or equal to 80, or infratentorial location did not influence in-hospital mortality for either race based on this multivariable model. Notably, the influence of age on mortality among Black patients was attenuated in the multivariable model after adjustment for hyperlipidemia and antiplatelet use. The ROC curves demonstrated an area under the curve (AUC) of about 0.9, indicating that these models performed well overall. Comparing a model of the original ICH score components (Fig. 2) to our new model including the additional components (Fig. 3), the AUC increased by 0.04 for Black ICH patients and 0.011 for White ICH patients.

**Table 4.** Multivariable analysis of in-hospital mortality by race

	Black patients (*n* = 114)			White patients (*n* = 161)		
Variable	OR	95% CI	*P*-value	OR	95% CI	*P*-value
Age ≥ 80	1.86	0.183–18.8	0.601	2.59	0.63–10.7	0.188
GCS categories, per one category point (e.g., 13–15 = 0 pts, 5–12 = 1 pt, 3–4 = 2 pts)	2.69	0.75–9.64	0.130	13.0	3.74–45.3	<0.0001
Initial hematoma volume, per 1 mL	1.05	1.01–1.09	0.029	1.04	1.01–1.06	<0.01
Intraventricular hemorrhage	3.36	0.51–22.3	0.208	1.89	0.57–6.25	0.297
Infratentorial location	16.5	0.72–376.0	0.079	1.26	0.19–8.57	0.812
Hyperlipidemia	13.9	1.54–127.0	0.019	1.14	0.28–4.56	0.855
Antiplatelet use	1.97	0.33–11.6	0.896	2.48	0.52–11.9	0.256
Admission leukocyte count, per 1.0 × 10^3^ per µL	1.06	0.86–1.31	0.572	1.14	0.97–1.34	0.208
Admission glucose level, per 1 mg/dL	1.00	0.99–1.01	0.896	1.002	0.99–1.01	0.425

GCS, Glasgow Coma Scale.

### Predictors of Poor Functional Outcome

For both Black and White patients, poor functional outcome was predicted by admission NIHSS, ICH score, and initial hematoma volume (Supplemental Table 1). For Black patients only, history of hypertension, antiplatelet use, and intubation status was significantly associated with functional outcomes. The association of hypertension with functional outcomes remained significant after controlling for initial hematoma volume. Also, for Black patients only, interhospital transfer was predictive of good functional outcomes. For White patients only, prior stroke or TIA, admission leukocyte count, admission glucose, neurosurgical intervention (EVD placement), intraventricular extension, and presence of microhemorrhages were each independently significantly associated with poor functional outcome.

## Discussion

The purpose of this study was to determine risk factors, ICH characteristics, and outcomes by race for ICH patients within the US Stroke Belt. The overall mortality was determined to be 30% with no differences in ICH severity, mortality, or discharge mRS between Black and White patients. This was true despite smaller initial hematoma volumes among Black patients. Age was associated with ICH etiology and location, and this association was present for both Black and White ICH patients. The ICH score, initial hematoma volume, intraventricular extension, intubation, and higher initial glucose or leukocyte count were consistently predictive of poor outcomes for both races. Intubation was a particularly strong predictor of in-hospital mortality. We noted that clinical documentation of hyperlipidemia (admission total cholesterol) and admission antiplatelet use were predictors of in-hospital mortality for only Black ICH patients. We also observed an increase in code status changes to DNR among young Black patients compared to young White patients. Finally, in examining a race-specific ICH score, we found hyperlipidemia to be a significant predictor for Black patients, but not White patients.

In our sample, Black ICH patients were on average more than 10 years younger than White ICH patients. This finding is consistent with prior literature [3, 21]. The generally younger age of Black patients with ICH may be related to differences in hemorrhage etiology suggested by the more frequent history of hypertension among our Black patients. Previously, the REGARDS study demonstrated that Black participants were more aware of their hypertension than White participants but less likely to have their blood pressure controlled [22]. Prior studies have also shown that hypertension is a powerful risk factor for spontaneous ICH for Black and White patients [7], with a higher risk for Black patients. Taken together, these two studies emphasize the importance of addressing this disparity. Furthermore, although the history of hypertension was not associated with in-hospital mortality, it was independently associated with poor functional outcomes at discharge for Black patients.

Hypertension seemed to play a similar role for both Black and White ICH patients, consistent with the known associations of hypertensive etiology and deep location with young age [23]. When first compared solely on the basis of age (not race), young patients had higher rates of deep bleeds and etiologies determined to be hypertensive or cryptogenic. Yet, further stratification by both race and age did not reveal significant differences in ICH location, etiology, outcome, or rates of intraventricular hemorrhage across races and ages. Nevertheless, smaller initial hematoma volumes were seen among young Black ICH patients compared to young White ICH patients. One potential confounder is the increased interhospital transfer rate among young White patients compared to young Black patients. It is possible that interhospital transfer may be associated with early hematoma growth due to delays in the provision of acute hemorrhage care including blood pressure management, neurosurgical intervention, and neurocritical care.

Within this sample, we failed to show that age differences significantly affected outcomes by race. Older age has been associated with ICH etiology [12] and lobar location [12, 13], increased hematoma volume and intraventricular hemorrhage [13], as well as poor outcome [24]. However, it has also been shown that advanced age and cerebral atrophy may offer some protection against in-hospital mortality after ICH [25], perhaps due to a greater room for hematoma expansion. In our study, as only some of these associations were seen by age stratification alone, the further stratification by both race and age may have limited our statistical power in examining this topic.

Our study did not identify differences in poor outcomes based on sex, which contrasts with the Ethnic and Racial Variations in Intracerebral Hemorrhage (ERICH) study’s report that women had a poorer functional outcome based on discharge mRS [26]. Our study also did not find differences in markers of ICH severity (initial hematoma volumes, ICH scores) based on sex. This further supports the notion that our ICH cases were similar among both sexes. Nevertheless, as clear differences based on sex in both mortality and poor functional outcome were noted at 90 days in ERICH, our study’s inability to support this finding may be due to the limited follow-up in our study.

We also noted an increase in the rates of code status change to DNR for young Black patients compared to young White patients, though no difference was noted in the older age groups. This finding supports results from ERICH suggesting that differences in goals of care decision-making may exist among ICH patients by race [27], as well as other studies showing Black patients were less likely to have DNR changes after subarachnoid hemorrhage [28] and younger patients were less likely to have DNR changes [29]. As no differences in in-hospital mortality or poor discharge functional outcome were seen here based on race (similar to ERICH), the difference in changes to DNR status between races warrants further investigation. While our finding potentially indicates differences in the quality of end-of-life care, the specific use of certain comfort measures was not examined here. Future studies should examine this topic in greater detail among ICH patients by race.

The effect of antiplatelet therapy on ICH outcomes continues to be a contested topic. In our study, documented antiplatelet use on admission was an independent predictor of in-hospital mortality for Black ICH patients, but not White ICH patients. Some prior studies have clearly shown an association of documented antithrombotic use with increased hematoma growth [30] and ICH mortality [31], and others have only shown this association with pre-ICH use of combination antiplatelet therapy [32]. Still, others have continued to demonstrate that the use of antiplatelets after ICH did not increase the risk of recurrent ICH [33], higher hematoma volumes, subsequent expansion, or 90-day clinical outcomes [34].

The ICH score has been tested in specific subpopulations, such as Argentinian or Asian populations [35, 36], but further validation specifically for Black patients is still needed. To address this, we analyzed a race-specific ICH score for predicting in-hospital mortality, as has been done in a prior study by Faigle *et al.* [37]. ICH characteristics were similar by race to the prior study, with lower initial hematoma volumes and lower median ICH scores among Black patients. GCS was predictive for only White patients in our study, as opposed to being predictive for either race in the prior study. In our sample, accounting for hyperlipidemia increased the association of this model with eventual in-hospital mortality for Black patients from ICH. Overall, both the study by Faigle *et al.* and our study demonstrated that the ICH score’s effectiveness may differ by race. As both age and etiology may play a significant role in ICH outcomes, the increased proportion of ICH among young Black ICH patients continues to indicate that race-specific validation of this tool on a larger scale may be needed.

Prior literature regarding hyperlipidemia as a risk factor for an ICH event has been mixed [38]. Few studies have considered whether hyperlipidemia increases the risk for in-hospital mortality or poor functional outcome after ICH. While it is true that hyperlipidemia may serve here as a surrogate marker for other unknown factors, its continued significance in the multivariable model after controlling for other variables makes this less likely to be the case. Hyperlipidemia has particularly been associated with nonlobar ICH, and Black patients suffer from nonlobar ICH at increased rates [39]. The significance of hyperlipidemia to ICH outcomes among Black patients needs further examination.

Our study has several strengths including providing details on ICH patients from a representative sample in the US Stroke Belt with a high proportion of Black patients, which allowed for stronger statistical comparisons with White ICH patients. We collected variables to define predictors of in-hospital mortality and discharge functional status with considerations to age and race among a high-risk patient population. Besides these strengths, there are several limitations that must be considered. First, our sample is based on a single-site location at a tertiary stroke center, which may limit generalizability, particularly outside the Stroke Belt and at nonacademic centers. Although the sample was ascertained in a prospective manner to allow for complete capture of all ICH patients presenting to our center, the data itself was ultimately abstracted in a retrospective fashion. Also, the method of defining past medical history as a dichotomous variable may have limited its accuracy. A potential alternative for improving this limitation is using multiple pilot tests to test the study coding manual [40]. This may lead to increased internal validity and reproducibility. Yet, even with the use of a coding manual, inaccuracies for this data may be due to variations in clinical documentation methods among healthcare providers, not necessarily as a result of data abstraction methodology. Furthermore, a high proportion of our sample was received in interhospital transfer, and we did not have access to medical records from external facilities. Finally, the primary endpoints of this study involved short-term outcomes precluding differences in outcomes that may become more apparent with longer follow-up. Despite these limitations, this study addresses a number of questions for ICH disparities in the Stroke Belt, as well as topics relevant to in-hospital assessment and prognostication for ICH.

## Conclusion

In a sample of ICH patients in a high-risk population in the Southeast USA, we observed a number of biracial differences in risk factor prevalence, patient demographics, and ICH characteristics. We confirmed prior observations of increased prevalence of hypertension, younger age, and increased rates of code status change to DNR among Black stroke patients. We also demonstrated novel disparities involving hyperlipidemia, admission antiplatelet use, and an improved race-specific ICH score. Aside from these observations, in-hospital mortality and immediate functional outcomes were similar among Black and White ICH patients. The observed biracial disparities in ICH related to prevalence of risk factors, differences in the care given to those suffering from non-survivable injuries, and relevance of prognostic tools such as the ICH score point to areas of potential improvement in ICH care.

## Supplementary material

For supplementary material accompanying this paper visit http://dx.doi.org/10.1017/cts.2021.21. click here to view supplementary material
