# Race and in-hospital mortality after spontaneous intracerebral hemorrhage in the Stroke Belt: Secondary analysis of a case-control study – CORRIGENDUM

**DOI:** 10.1017/cts.2022.2

**Published:** 2022-02-03

**Authors:** Logan D. Hilton, Michael J. Lyerly, Toby I. Gropen

**Keywords:** Spontaneous intracerebral hemorrhage, stroke, ICH, race, disparities, corrigendum

The above article [1] published with two errors in the Discussion section and one error in the Conclusion section. These errors incorrectly noted an increase in change to DNR status among young Black patients when compared to young White patients.

On page 6, right-hand column, the sixth full sentence should read “We also observed a decrease in code status changes to DNR among young Black patients compared to young White patients.”

On page 9, left-hand column, the first sentence of the second full paragraph should read “We also noted a decrease in the rates of code status change to DNR for young Black patients compared to young White patients, though no difference was noted in the older age groups.”

On page 9, right-hand column, the second sentence in the Conclusion section should read “We confirmed prior observations of increased prevalence of hypertension, younger age, and decreased rates of code status change to DNR among Black stroke patients.”

The authors apologize for this error.
