# Supplementary material for: Race and in-hospital mortality after spontaneous intracerebral hemorrhage in the Stroke Belt: Secondary analysis of a case–control study
Source: J Clin Transl Sci. 2021 Mar 16;5(1):e115. doi: 10.1017/cts.2021.21 (PMC8223176; doi:10.1017/cts.2021.21)
Supplement: Supplementary file 1 [file S2059866121000212sup001.docx]

Supplemental Table 1: Predictors of Poor Functional Outcomes by Discharge mRS

|  | Blacks  (n=114) | | | Whites  (n=161) | | | |
| --- | --- | --- | --- | --- | --- | --- | --- |
| **Variable** | OR | 95% CI | p-value | OR | 95% CI | p-value |  |
| Age, per year | 1.03 | 0.996-1.06 | 0.09 | 1.00 | 0.977-1.026 | 0.91 |  |
| Sex, Male | 0.83 | 0.36-1.90 | 0.65 | 0.8 | 0.40-1.60 | 0.53 |  |
| Geography, Rural | 1.27 | 0.42-3.80 | 0.67 | 0.69 | 0.34-1.40 | 0.31 |  |
| Interhospital Transfer | **0.28** | **0.12-0.67** | **<0.01** | 0.52 | 0.22-1.24 | 0.14 |  |
| **Comorbidities** | | | | | | | |
| Prior Stroke/TIA | 1.51 | 0.55-4.16 | 0.43 | **8.29** | **1.89-36.2** | **<0.01** |  |
| HTN | **3.27** | **1.04-10.3** | **0.04** | 1.19 | 0.56-2.54 | 0.65 |  |
| DM | 2.72 | 0.86-8.70 | 0.09 | 1.93 | 0.81-4.59 | 0.14 |  |
| HLD | 2.94 | 0.80-10.8 | 0.10 | 0.81 | 0.37-1.77 | 0.60 |  |
| Depression | 1.52 | 0.30-7.62 | 0.61 | 1.65 | 0.34-8.07 | 0.54 |  |
| Dementia | 3.04 | 0.36-25.5 | 0.31 | 2.57 | 0.55-11.9 | 0.23 |  |
| Psychiatric Illness | 1.4 | 0.36-5.43 | 0.63 | 1.83 | 0.49-6.77 | 0.36 |  |
| **Medications** | | | | | | | |
| Antiplatelet Use | **3.61** | **1.15-11.3** | **0.03** | 1.26 | 0.57-2.78 | 0.57 |  |
| Anticoagulant Use | 0.92 | 0.30-2.83 | 0.88 | 1.29 | 0.51-3.29 | 0.58 |  |
| Statin Use | 1.89 | 0.69-5.18 | 0.21 | 1.17 | 0.53-2.59 | 0.69 |  |
| **Lab Values** | | | | | | | |
| Admit WBC Count | 1.09 | 0.95-1.25 | 0.23 | **1.13** | **1.03-1.24** | **0.01** |  |
| Admit Platelet Count, per 1.0 x 10^9^/L | 1.00 | 0.99-1.01 | 0.97 | 1.00 | 0.996-1.00 | 0.97 |  |
| Admit Glucose, per 1 mg/dL | 1.007 | 0.99-1.02 | 0.14 | **1.02** | **1.01-1.03** | **<0.001** |  |
| Admit PT | 1.02 | 0.95-1.11 | 0.54 | 1.04 | 0.95-1.15 | 0.40 |  |
| Admit INR | 1.25 | 0.63-2.51 | 0.52 | 1.46 | 0.59-3.64 | 0.41 |  |
| Admit Total Chol, per 1 mg/dL | 0.99 | 0.99-1.01 | 0.84 | 0.99 | 0.98-1.001 | 0.08 |  |
| **Hospital Course** | | | | | | | |
| NIHSS, per 1pt | **1.19** | **1.09-1.30** | **<0.001** | **1.46** | **1.25-1.71** | **<0.0001** |  |
| ICH score, per 1pt | **2.18** | **1.38-3.46** | **<0.001** | **2.92** | **1.91-4.45** | **<0.0001** |  |
| Intubation | **6.75** | **1.9-24.0** | **<0.01** | >999* |  | 0.94 |  |
| NSGY intervention | >999* |  | 0.95 | **5.32** | **1.19-23.6** | **0.03** |  |
| ICP Monitor | 3.3 | 0.71-15.3 | 0.13 | **4.75** | **1.07-21.2** | **0.04** |  |
| Changed to DNR | >999* |  | 0.97 | >999* |  | 0.96 |  |
| Reversal Therapy | 0.69 | 0.16-2.96 | 0.62 | 1.1 | 0.37-3.25 | 0.87 |  |
| **Radiologic Data** | | | | | | | |
| Initial Hematoma Vol, per 1mL | **1.06** | **1.02-1.10** | **<0.01** | **1.06** | **1.03-1.10** | **<0.001** |  |
| IVH | 2.27 | 0.91-5.68 | 0.08 | **6.06** | **2.59-14.2** | **<0.0001** |  |
| Spot Sign, if CTA | 2.95 | 0.34-25.7 | 0.32 | >999* |  | 0.95 |  |
| Hematoma Expansion | >999* |  | 0.96 | 6.00 | 0.75-47.9 | 0.09 |  |
| Location, Lobar | 0.64 | 0.27-1.51 | 0.31 | 0.95 | 0.47-1.95 | 0.89 |  |
| Location, Deep | 1.77 | 0.74-4.25 | 0.20 | 1.37 | 0.67-2.79 | 0.39 |  |
| Microhemorrhages | 1.58 | 0.61-4.06 | 0.35 | **2.32** | **1.02-5.27** | **0.045** |  |
| Deep | 2.75 | 0.95-7.98 | 0.06 | 1.98 | 0.81-4.85 | 0.13 |  |
| Cortical | 0.39 | 0.12-1.22 | 0.11 | 2.39 | 0.87-6.59 | 0.09 |  |
| >10 | 0.83 | 0.30-2.36 | 0.73 | 2.07 | 0.79-5.44 | 0.14 |  |
| Superficial Siderosis | 0.32 | 0.05-2.05 | 0.23 | 5.25 | 0.64-43.2 | 0.12 |  |
| *Complete association of variable with poor discharge mRS | | | | | | |  |
